# pH-Induced Association and Dissociation of Intermolecular Complexes Formed by Hydrogen Bonding between Diblock Copolymers

**DOI:** 10.3390/polym9080367

**Published:** 2017-08-17

**Authors:** Masanobu Mizusaki, Tatsuya Endo, Rina Nakahata, Yotaro Morishima, Shin-ichi Yusa

**Affiliations:** 1 Department of Applied Chemistry, Graduate School of Engineering, University of Hyogo, 2167 Shosha, Himeji, Hyogo 671-2280, Japan; ta-endou@toyo-rubber.co.jp (T.E.); nkht9999@gmail.com (R.N.); 2 Faculty of Engineering, Fukui University of Technology, 6-3-1 Gakuen, Fukui 910-8505, Japan; morisima@fukui-ut.ac.jp

**Keywords:** block copolymers, RAFT polymerization, complex, hydrogen bonding interactions, pH-responsive

## Abstract

Poly(sodium styrenesulfonate)–*block*–poly(acrylic acid) (PNaSS–*b*–PAA) and poly(sodium styrenesulfonate)–*block–*poly(*N*-isopropylacrylamide) (PNaSS–*b–*PNIPAM) were prepared via reversible addition–fragmentation chain transfer (RAFT) radical polymerization using a PNaSS-based macro-chain transfer agent. The molecular weight distributions (*M*_w_/*M*_n_) of PNaSS–*b*–PAA and PNaSS–*b–*PNIPAM were 1.18 and 1.39, respectively, suggesting that these polymers have controlled structures. When aqueous solutions of PNaSS–b–PAA and PNaSS–*b–*PNIPAM were mixed under acidic conditions, water-soluble PNaSS–*b–*PAA/PNaSS–*b–*PNIPAM complexes were formed as a result of hydrogen bonding interactions between the pendant carboxylic acids in the PAA block and the pendant amide groups in the PNIPAM block. The complex was characterized by ^1^H NMR, dynamic light scattering, static light scattering, and transmission electron microscope measurements. The light scattering intensity of the complex depended on the mixing ratio of PNaSS–*b*–PAA and PNaSS–*b–*PNIPAM. When the molar ratio of the *N*-isopropylacrylamide (NIPAM) and acrylic acid (AA) units was near unity, the light scattering intensity reached a maximum, indicating stoichiometric complex formation. The complex dissociated at a pH higher than 4.0 because the hydrogen bonding interactions disappeared due to deprotonation of the pendant carboxylic acids in the PAA block.

## 1. Introduction

Non-covalent interactions, such as hydrophobic [1], electrostatic [2,3], van der Waals [4], and hydrogen bonding interactions [5,6], can be a driving force for the complex formation of polymers. In particular, hydrogen bonding interactions are an important driving force for the self-organization of natural polymers such as polysaccharides, proteins, and deoxyribonucleic acid (DNA).

It is known that hydrogen bonding interactions between amide groups or poly(ethylene glycol) (PEG) and carboxylic acid groups promote self-association or complex formation in water [7,8,9]. Shieh et al. [10] prepared a series of poly(*N*-isopropylacrylamide-*random*-acrylic acid) (P(NIPAM–*r–*AA)) copolymers and examined their glass transition behavior. They found that the incorporation of acrylic acid units into the PNIPAM polymer enhances the glass transition temperature (*T*_g_) due to intermolecular hydrogen bonding between the pendant isopropyl amide groups and the carboxylic acid groups using ^1^H NMR and Fourier-transform infrared analyses. As an example of such complex formation, Bian et al. [11] investigated the interaction between poly(*N*,*N*-diethylacrylamide) (PDEA), which is an analog of PNIPAM, and poly(acrylic acid) (PAA). The complex is formed between the two polymers through hydrogen bonding interactions with a stoichiometry of *r* = 0.6 (*r* is the unit molar ratio of PAA/PDEA), and the complex formation depends on pH values. We lately reported [12] the complex formation behavior of poly(sodium stylenesulfonate)–*block–*PEG–*block–*poly(sodium stylenesulfonate) (PNaSS–*b*–PEG–*b*–PNaSS) with poly(methacrylic acid) (PMA). Both PNaSS–*b–*PEG–*b*–PNaSS and PMA were synthesized via reversible addition–fragmentation chain transfer (RAFT) radical polymerization. Below pH 5, water-soluble complexes were formed owing to the hydrogen bonding interactions between the PEG block in PNaSS–*b*–PEG–*b*–PNaSS and the carboxylic acids in PMA. The experimental data indicated that the PNaSS–*b*–PEG–*b*–PNaSS/PMA complex was spherical in shape. At pH greater than 5, the complex dissociated because the hydrogen bonding interaction disappeared due to deprotonation of the pendant carboxylic acids in PMA.

**Figure 1:**
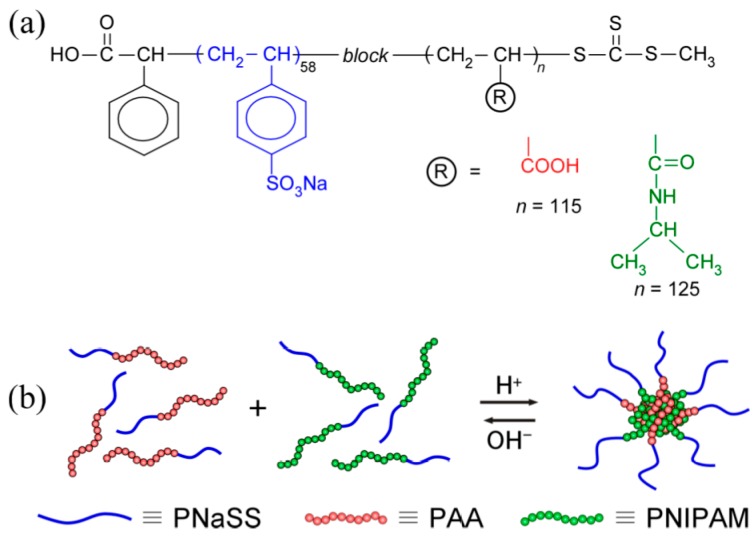
(**a**) Chemical structures of diblock copolymers used in this study: Poly(sodium styrenesulfonate)_58_–*block*–poly(acrylic acid)_125_ (PNaSS_58_–*b*–PAA_125_) and poly(sodium styrenesulfonate)_58_–*block*–poly(*N*-isopropylacrylamide)_115_ (PNaSS_58_–*b*–PNIPAM_115_), and (**b**) schematic representation of polymer chain mixing in the core and corona micelles comprising PNaSS_58_–*b*–PAA_125_ and PNaSS_58_–*b*–PNIPAM_115_.

In the present study, we focused on the complex formation behavior owing to the hydrogen bonding interactions as a function of the solution temperature instead of the solution pH. Two species of diblock copolymers (Figure 1a), PNaSS–*block*–poly(acrylic acid) (PNaSS–*b*–PAA) and PNaSS–*block*–PNIPAM (PNaSS–*b*–PNIPAM), were synthesized via RAFT radical polymerization [13]. The PNIPAM blocks of PNaSS-*b*-PNIPAM are also assumed to associate above the lower critical solution temperature (LCST) to form core–corona-type multi-polymer micelles [14]. When PNaSS–*b*–PAA and PNaSS–*b*–PNIPAM were mixed in a 0.1 M NaCl aqueous solution at 20 °C, water-soluble PNaSS–*b*–PAA/PNaSS–*b*–PNIPAM complexes were formed through hydrogen bonding interactions between the PAA and PNIPAM blocks below pH 3.9 (Figure 1b). The complexes were maintained above the LCST of PNaSS–*b*–PNIPAM. The complexes dissociated under basic conditions when the hydrogen bonding interactions disappeared due to deprotonation of the pendant carboxylic acids in the PAA block.

## 2. Experimental Section

### 2.1. Materials 

Acrylic acid (AA) from Kanto Chemical (Tokyo, Japan) was dried over 4 Å molecular sieves and distilled under reduced pressure. *N*-isopropylacrylamide (NIPAM) from Aldrich (St. Louis, MO, USA) was purified by recrystallization from a mixed solvent of benzene and *n*-hexane. α-Methyltrithiocarbonate-*S*-phenylaceticacid (MTPA) was synthesized as previously reported [15]. Sodium styrenesulfonate (NaSS) from Tokyo Chemical Industry (Tokyo, Japan) and 4,4′-azobis(4-cyanopentanoic acid) (V-501) from Aldrich were used as received. Methanol was dried using molecular sieves and distilled. Deionized water was used.

### 2.2. Synthesis of PNaSS Macro-Chain Transfer Agent (PNaSS Macro–CTA)

NaSS (20.6 g, 100 mmol) and MTPA (0.26 g, 1.0 mmol) were dissolved in 180 mL of water, and V-501 (5.6 mg, 0.2 mmol) was added to the aqueous solution. Polymerization was carried out at 70 °C for 3 h under Ar atmosphere. After the polymerization, the mixture was dialyzed against pure water for a week and recovered by a freeze-drying technique (yield 14.8 g, number-average molecular weight (*M*_n_) = 1.22 × 10^4^ (gel permeation chromatography (GPC)), molecular weight distribution (*M*_w_/*M*_n_) = 1.19, and degree of polymerization (DP) = 58). The obtained PNaSS could be used as a macro-CTA (PNaSS_58_ macro–CTA).

### 2.3. Preparation of PNaSS_58_–b–PAA_125_

AA (0.86 g, 12 mmol) was dissolved in 15 mL of water, and PNaSS_58_ macro–CTA (1.05 g, 0.087 mmol) and V-501 (5.0 mg, 0.018 mmol) were added to this solution. The mixture was deoxygenated by purging with Ar gas for 30 min. Block copolymerization was carried out at 70 °C for 2 h. The diblock copolymer was purified by dialysis against pure water for a week and then recovered using a freeze-drying technique. The diblock copolymer, PNaSS_58_–*b–*PAA_125_, was obtained (yield 1.79 g, *M*_n_ = 2.12 × 10^4^ (^1^H NMR), *M*_w_/*M*_n_ = 1.18, and DP of the PAA block = 125).

### 2.4. Preparation of PNaSS_58_–b–PNIPAM_115_

NIPAM (2.26 g, 10.2 mmol) was dissolved in a mixed solvent of water and methanol (12.4 mL, 1/1, *v/v*), and PNaSS_58_ macro–CTA (0.96 g, 0.079 mmol) and V-501 (4.6 mg, 0.016 mmol) were added to this solution. The mixture was deoxygenated by purging with Ar gas for 30 min. Block copolymerization was carried out at 60 °C for 5 h. The diblock copolymer was purified by reprecipitation from a methanol solution into excess ether twice and then recovered using a freeze-drying technique. The diblock copolymer PNaSS_58_–*b*–PNIPAM_115_ was obtained (yield 1.79 g, *M*_n_ = 2.52 × 10^4^ (^1^H NMR), *M*_w_/*M*_n_ = 1.39, and DP of the PNIPAM block = 115).

### 2.5. Preparation of the Water-Soluble Complex

Stock solutions of PNaSS_58_–*b*–PAA_125_ and PNaSS_58_–*b*–PNIPAM_115_ were prepared by dissolving each polymer in 0.1 M NaCl aqueous solutions of pH 3 and 10, respectively. To prepare the complex, the PNaSS_58_–*b*–PNIPAM_115_ aqueous solution was added to the PNaSS_58_–*b*–PAA_125_ aqueous solution over a period of five min, and the mixture was allowed to stand still for one day. The mixing ratio of the two diblock copolymers was adjusted based on the molar fraction of NIPAM units (*f*_NIPAM_ = (NIPAM)/((NIPAM) + (AA)), where (NIPAM) and (AA) are the molar concentrations of NIPAM and AA units, respectively). The complex was prepared at *f*_NIPAM_ = 0.5 unless otherwise noted.

### 2.6. Measurements

GPC measurements were performed with a Shodex 7.0 μm bead size GF-7F HQ column. A phosphate buffer at pH 9, containing 10 vol % acetonitrile was used as an eluent at a flow rate of 0.6 mL/min at 40 °C. *M*_n_ and *M*_w_/*M*_n_ were calibrated with standard PNaSS samples of 11 different molecular weights ranging from 1.37 × 10^3^ to 2.61 × 10^6^. ^1^H NMR spectra were obtained with a Bruker DRX-500 spectrometer (Buick Rica, MA, USA) operating at 500 MHz. Light scattering measurements were performed using an Otsuka Electronics Photal DLS-7000DL equipped with a digital time correlator (ALV-5000E, Osaka, Japan). Sample solutions were filtered with a 0.2-μm membrane filter. For dynamic light scattering (DLS) measurements, the data obtained were analyzed with ALV software version 3.0 [16,17]. For static light scattering (SLS) measurements, weight-average molecular weight (*M*_w_), and radius of gyration (*R*_g_) were estimated from Zimm plots [18]. Values of *dn/dC_P_* were determined with an Otsuka Electronics Photal DRM-1020 differential refractometer. Transmission electron microscopy (TEM) measurements were carried out using a JEOL JEM-2100 (Tokyo, Japan) at an accelerating voltage of 200 kV. The TEM sample was prepared by placing one droplet of the solution on a copper grid coated with Formvar. The sample was stained by sodium phosphotungstate and dried under reduced pressure. Percent transmittance (%*T*) measurements were performed using a JASCO V-630 BIO UV–Vis spectrometer (Tokyo, Japan) with a 10 mm path length quartz cell. The temperature was increased from 20 to 80 °C with a heating rate of 1.0 °C·min^−1^ using a JASCO ETC-717 thermostat system.

## 3. Results and Discussion

We prepared the diblock copolymers, PNaSS_58_–*b*–PAA_125_ and PNaSS_58_–*b*–PNIPAM_115_, via a RAFT technique using PNaSS_58_ macro–CTA. The DP of PNaSS_58_ macro–CTA, which was determined by GPC, was 58 (PNaSS_58_–macro–CTA). The DPs of the PAA and PNIPAM blocks were 125 and 115, respectively. These values were calculated from ^1^H NMR peak area intensities derived from the PAA or PNIPAM block and an area derived from the PNaSS block. *M*_n_ and *M*_w_/*M*_n_ of the diblock copolymers were estimated from GPC. The results for the characteristics of the diblock copolymers are listed in Table 1. The *M*_w_/*M*_n_ values are relatively small (*M*_w_/*M*_n_ < 1.4), indicating that the controlled/living polymerizations proceeded successfully [13].

When the polymerization is assumed an ideally living process, then the theoretical number-average molecular weight (*M*_n_(theo)) can be estimated as

(1) $$M_{n}\left(\mathrm{theo}\right)=\frac{{\left[M\right]}_{0}\;x_{m}}{{\left[\mathrm{CTA}\right]}_{0}\;100}M_{m}+M_{\mathrm{CTA}}$$

where [M]_0_ is the initial monomer concentration, [CTA]_0_ is the initial PNaSS_58_ macro–CTA concentration, *x*_m_ is the conversion of the monomer, *M*_m_ is the molecular weight of the monomer, and *M*_CTA_ is the molecular weight of PNaSS_58_ macro–CTA. The *M*_n_(NMR) values for PNaSS_58_–*b*–PAA_125_ and PNaSS_58_–*b*–PNIPAM_115_ were calculated from the ^1^H NMR data. As shown in Table 1, the *M*_n_(NMR) values for PNaSS_58_–*b*–PAA_125_ and PNaSS_58_–*b*–PNIPAM_115_ were in reasonable agreement with the *M*_n_(theo) values. However, the *M*_n_(theo) and *M*_n_(GPC) values for both diblock copolymers were found to be slightly different. This may be because the volume-to-mass ratio for PNaSS is different from those for SS_58_–*b*–PAA_125_ and PNaSS_58_–*b*–PNIPAM_115_ [19,20].

**Figure 2:**
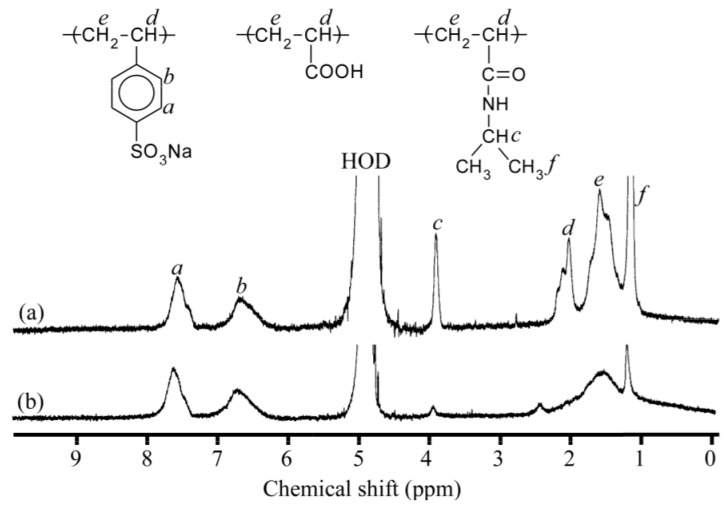
^1^H NMR spectra and peak assignment of the mixture of PNaSS_58_–*b*–PAA_125_ and PNaSS_58_–*b*–PNIPAM_115_ in D_2_O containing 0.1 M NaCl at (**a**) pH 10 and (**b**) pH 3.

We attempted to monitor complex formation between PNaSS_58_–*b*–PAA_125_ and PNaSS_58_–*b*–PNIPAM_115_ induced by the solution pH using ^1^H NMR spectra measured in D_2_O containing 0.1 M NaCl at pH 10 and 3. Figure 2 compares the ^1^H NMR spectra of mixed solutions at pH 10 and 3. In Figure 2a, the resonance peaks at 1.16 (*f*) and 3.91 ppm (*c*) are attributed to the methyl and methine protons, respectively, in the pendant isopropyl group of the PNIPAM block are observed. Moreover, the resonance bands owing to the pendant phenyl protons in the PNaSS block are also detected at 6.1–7.9 ppm (*a* and *b*). The resonance bands in the 1.28–2.38 ppm region (*d* and *e*) are attributed to the sum of the main chain of the diblock copolymers. At pH 3, the intensities of the resonance peaks *f* and *c* derived from the PNIPAM block remarkably decreased, whereas those of *a* and *b* remained intact, as shown in Figure 2b. Besides this result, the resonance bands in the 1.28–2.38 ppm region (*d* and *e*) became broad. From the significant reduction of the motional freedom for the PNIPAM block at pH 3, one can predict that the complexes are formed from PNaSS_58_–*b*–PAA_125_ and PNaSS_58_–*b*–PNIPAM_115_ owing to the hydrogen bonding interactions between the pendant carboxylic acids in the PAA block and the amide groups in the PNIPAM block. At pH 10, however, the complexes dissociated owing to the disappearance of the hydrogen bonding interactions as a result of deprotonation of the carboxylic acids in the PAA block.

**Figure 3:**
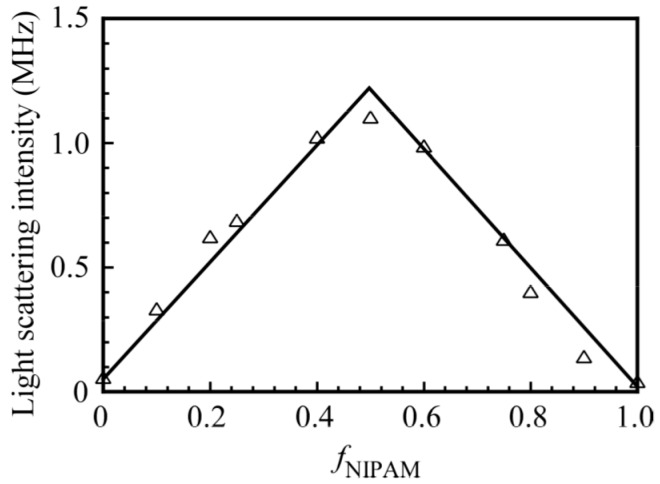
Light scattering intensities of the PNaSS_58_–*b*–PAA_125_/PNaSS_58_–*b*–PNIPAM_115_ complexes as a function of *f*_NIPAM_ (= (NIPAM)/((NIPAM) + (AA))) in 0.1 M NaCl at pH 3. The total polymer concentration was fixed at 4.4 g/L.

Figure 3 shows the light scattering intensities for a mixture of PNaSS_58_–*b*–PAA_125_ and PNaSS_58_–*b*–PNIPAM_115_ in 0.1 M NaCl at pH 3 as a function of *f*_NIPAM_. The total polymer concentration was kept constant at 4.4 g/L. An increase in the scattering intensity suggests an increase in the size of the complex. The maximum scattering intensity was observed at *f*_NIPAM_ = 0.5. This result indicates that a stoichiometric interaction in the mixture of PNaSS_58_–*b*–PAA_125_ and PNaSS_58_–*b*–PNIPAM_115_ led to form a complex with largest aggregation number. The complex with *f*_NIPAM_ = 0.5 was studied unless otherwise stated.

**Figure 4:**
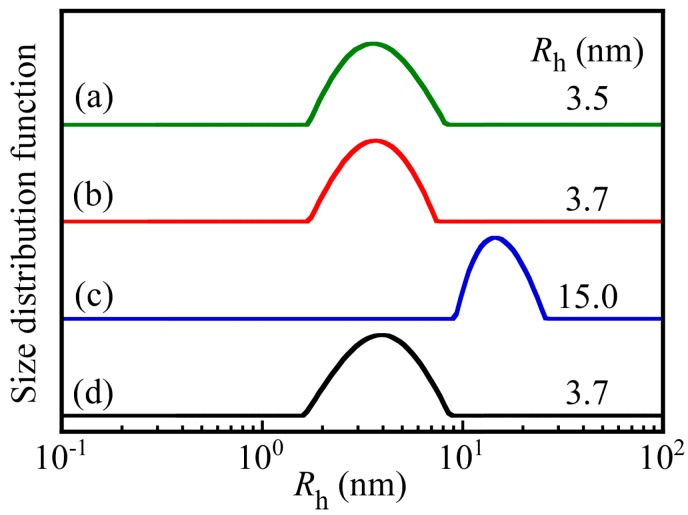
Hydrodynamic radius (*R*_h_) distributions for (**a**) PNaSS_58_–*b*–PAA_125_ and (**b**) PNaSS_58_–*b*–PNIPAM_115_ in 0.1 M NaCl at pH 3, and the PNaSS_58_–*b*–PAA_125_/PNaSS_58_–*b*–PNIPAM_115_ complex at (**c**) pH 3 and (**d**) pH 10.

Figure 4 shows the hydrodynamic radius (*R*_h_) distributions for each diblock copolymer and the PNaSS_58_–*b*–PAA_125_/PNaSS_58_–*b*–PNIPAM_115_ complex at pH 3 and 10. The values of *R*_h_ were determined by DLS in 0.1 M NaCl and are indicated in the figure. The *R*_h_ values for PNaSS_58_–*b*–PAA_125_ and PNaSS_58_–*b*–PNIPAM_115_ were 3.5 and 3.7 nm, respectively, which are reasonable for a unimer state. The *R*_h_ values of the complex at pH 3 and 10 were 15.0 and 3.7 nm, respectively. When PNaSS_58_–*b*–PAA_125_ and PNaSS_58_–*b*–PNIPAM_115_ were mixed at pH 3, a water-soluble complex was formed due to hydrogen bonding interactions. On the other hand, at pH 10 the complex dissociated because the hydrogen bonding interactions disappeared as a result of deprotonation of the pendant carboxylic acids.

**Figure 5:**
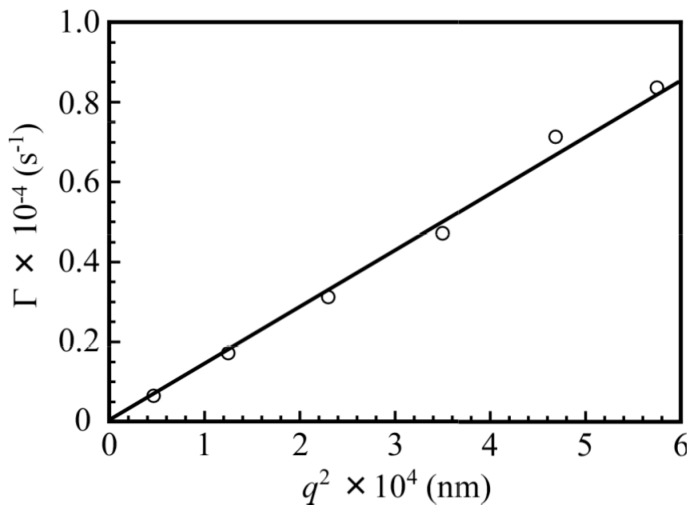
The relaxation rate (Γ) plotted as a function of the square of the magnitude of the scattering vector (*q*^2^) for the PNaSS_58_–*b*–PAA_125_/PNaSS_58_–*b*–PNIPAM_115_ complex at *C*_p_ = 4.4 g/L in 0.1 M NaCl at pH 3.

The relaxation rates (Γ) measured at different scattering angles (θ) are plotted as a function of the square of the magnitude of the scattering vector (*q*^2^) for the mixture of PNaSS_58_–*b*–PAA_125_ and PNaSS_58_–*b–*PNIPAM_115_ in 0.1 M NaCl at pH 3 in Figure 5. A linear plot passing through the origin suggests that the relaxation modes are virtually diffusive [21]. Thus, the *R*_h_ values can be estimated at a fixed θ of 90° as the angular dependence is negligible.

**Figure 6:**
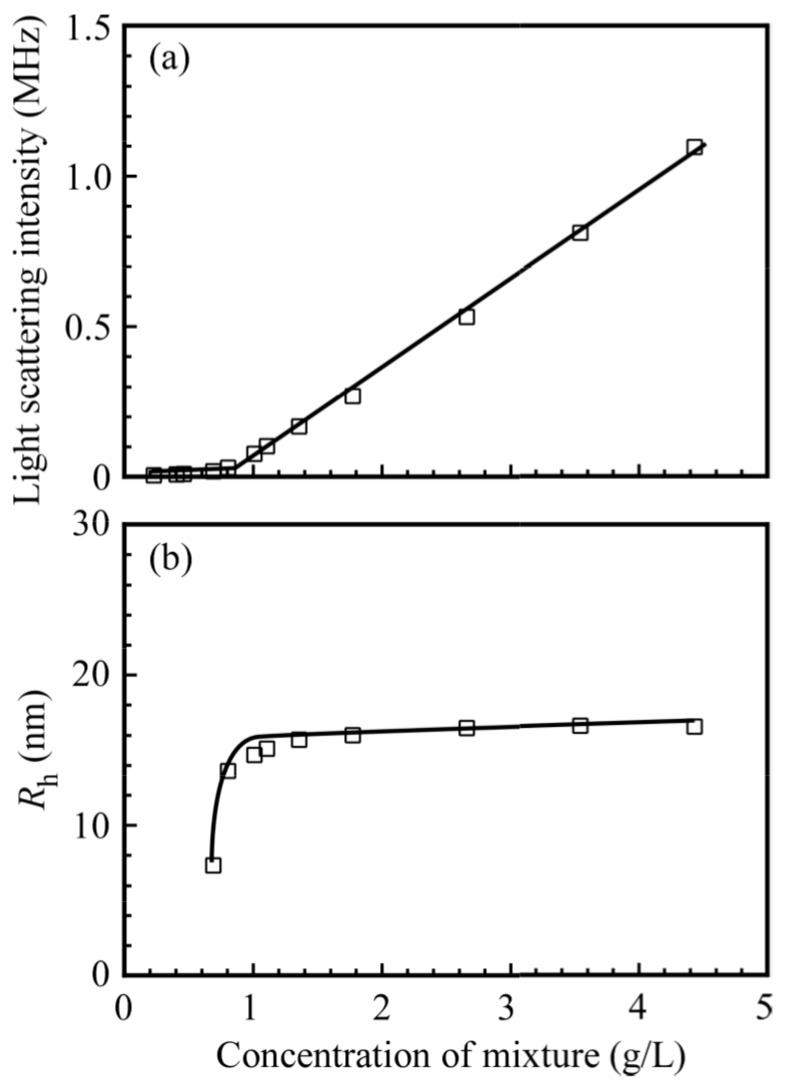
(**a**) Light scattering intensity and (**b**) hydrodynamic radius (*R*_h_) of the PNaSS_58_–*b–*PAA_125_/PNaSS_58_–*b*–PNIPAM_115_ complex as a function of the concentration of the mixture in 0.1 M NaCl at pH 3.

Figure 6 shows the light scattering intensities and *R*_h_ values for the mixture of PNaSS_58_–*b*–PAA_125_ and PNaSS_58_–*b*–PNIPAM_115_ in 0.1 M NaCl at pH 3 as a function of the concentration of the mixture. In Figure 6a, the scattering intensity increases sharply with increases in the concentration above a threshold of 0.9 g/L. Figure 6b indicates that the *R*_h_ values for the complex are practically constant (*R*_h_ is approximately 15 nm) in the concentration range from 0.9 to 4.4 g/L. The results suggest that the formation of the complex between PNaSS_58_–*b*–PAA_125_ and PNaSS_58_–*b*–PNIPAM_115_ starts to occur above a critical concentration of 0.9 g/L. The apparent critical aggregate concentration (CAC) value for the complex, estimated from the plots, is 0.9 g/L.

**Figure 7:**
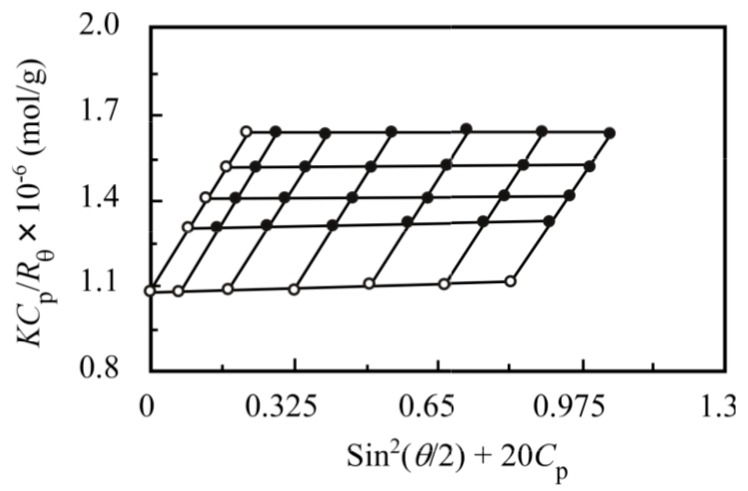
Zimm plot for the PNaSS_58_–*b*–PAA_125_/PNaSS_58_–*b*–PNIPAM_115_ complex with *f*_NIPAM_ = 0.5 in 0.1 M NaCl at pH 3. Scattering angles (θ) range from 30° to 130° in 20° increments.

The apparent values of *M*_w_ and *R*_g_, determined by SLS measurements, are listed in Table 2. Figure 7 shows a Zimm plot for the PNaSS_58_–*b*–PAA_125_/PNaSS_58_–*b*–PNIPAM_115_ complex, which is formed from a mixture of PNaSS_58_–*b*–PAA_125_ and PNaSS_58_–*b*–PNIPAM_115_ in 0.1 M NaCl at pH 3. The aggregation number (*N*_agg_) was defined as the number of polymer chains forming one complex, which can be estimated from the *M*_w_ values of the complex and unimer. The result of this calculation gives an *N*_agg_ of 46 for the complex. The chain numbers of PNaSS_58_–*b*–PAA_125_ and PNaSS_58_–*b*–PNIPAM_115_ for single complex are 22 and 24, respectively, as calculated from *f*_NIPAM_ = 0.5 and the DP values of PAA and PNIPAM.

The *R*_g_/*R*_h_ value indicates the shape of the molecular assemblies. The theoretical *R*_g_/*R*_h_ value of a homogeneous hard sphere is 0.778 but this value increases substantially for less dense structures and polydisperse mixtures; for example, *R*_g_/*R*_h_ = 1.5–1.7 for flexible linear chains in good solvents, whereas *R*_g_/*R*_h_ ≥ 2 for a rigid rod [22,23,24]. The *R*_g_/*R*_h_ ratio for the complex (Table 2) was 0.86, suggesting that the shape of the complex may be spherical. The *R*_g_/*R*_h_ ratio for the unimer was 3.7, which indicates that the unimer was in a relatively expanded conformation with polydispersity.

**Figure 8:**
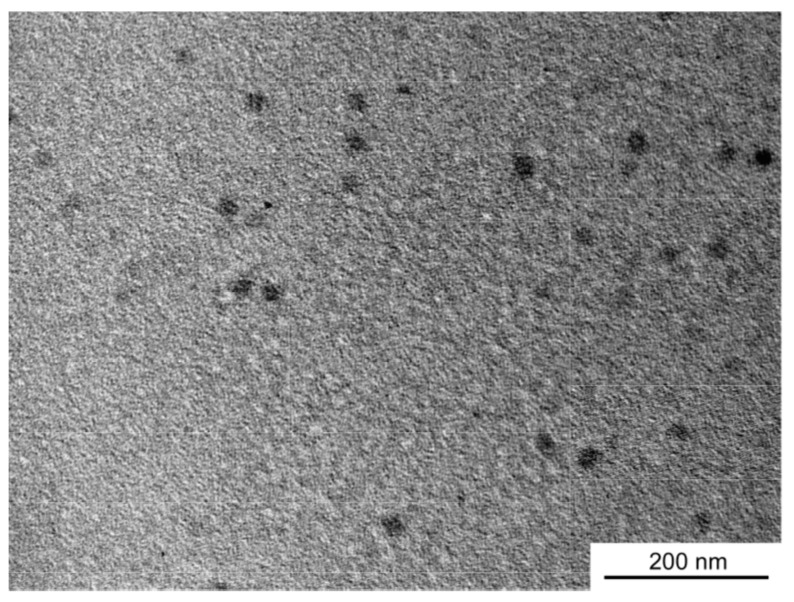
Transmission electron microscopy (TEM) image of the PNaSS_58_–*b*–PAA_125_/PNaSS_58_–*b*–PNIPAM_115_ complex at pH 3.

To confirm the shape and size of the PNaSS_58_–*b*–PAA_125_/PNaSS_58_–*b*–PNIPAM_115_ complex at pH 3, TEM measurements were performed (Figure 8). The complex formed spherical objects with almost uniform contrast, suggesting that it comprises micelles with PAA/PNIPAM cores and PNaSS shells. The average radius estimated from the TEM images for the complex was 13.4 nm, which is similar to the *R*_h_ value estimated from DLS.

**Figure 9:**
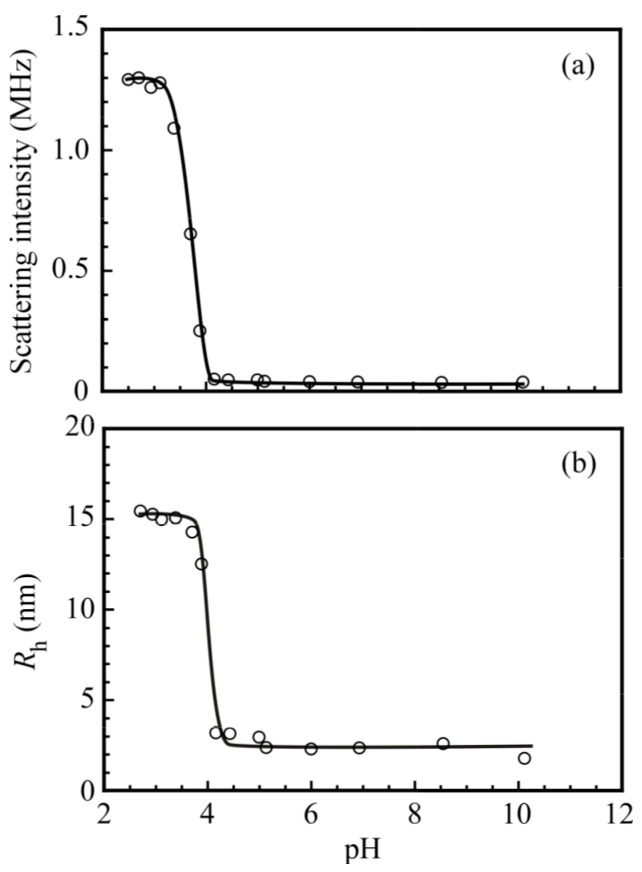
(**a**) Scattering intensity and (**b**) hydrodynamic radius (*R*_h_) of the PNaSS_58_–*b*–PAA_125_/PNaSS_58_–*b*–PNIPAM_115_ complex at *C*_p_ = 4.4 g/L in 0.1 M NaCl as a function of pH.

Figure 9a shows the light scattering intensities for the complex of PNaSS_58_–*b*–PAA_125_ and PNaSS_58_–*b*–PNIPAM_115_ in 0.1 M NaCl as a function of pH. The light scattering intensity increased rapidly as the pH decreased from 4.0 to 3.5, which suggests that the complex was formed below pH 3.5. The light scattering intensity was nearly constant between pH 3.5 and 2.5 suggests that *N*_agg_ was constant in this pH region because the light scattering intensity is proportional to the molecular mass. Figure 9b shows *R*_h_ values for the complex as a function of pH. Above pH 4.0, the *R*_h_ values were of the order of 3 nm, suggesting that PNaSS_58_–*b*–PAA_125_ and PNaSS_58_–*b*–PNIPAM_115_ were in a unimer state. As the pH decreased, *R*_h_ started to increase at around pH 4.0, reaching a maximum value of 15.0 nm at pH 3.5. As the pH continued to decrease, *R*_h_ was nearly constant between pH 3.5 and 2.5, suggesting that not the aggregation number but the compactness of the complex was practically constant.

**Figure 10:**
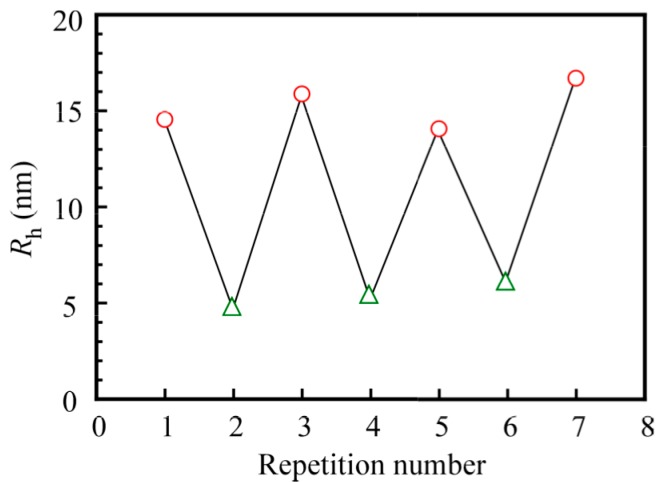
The hydrodynamic radius (*R*_h_) of the PNaSS_58_–*b*–PAA_125_/PNaSS_58_–*b*–PNIPAM_115_ complex at *C*_p_ = 4.4 g/L in 0.1 M NaCl at pH 3 (◯) and pH 10 (△).

When the pH value was increased from 3 to 10 and subsequently decreased back to 3, pH-induced *R*_h_ changes were found to be completely reversible. Figure 10 shows the pH-induced changes of the *R*_h_ value of the PNaSS_58_–*b*–PAA_125_/PNaSS_58_–*b*–PNIPAM_115_ complex in 0.1 M NaCl cycled between pH 3 and 10 with 20 min intervals. The changes in *R*_h_ between the two pH values were completely reproducible, indicating that the association and dissociation of the complex caused by pH changes was reversible over many cycles. This result suggests that the complex may find applications as a pH-responsive controlled association–dissociation system.

**Figure 11:**
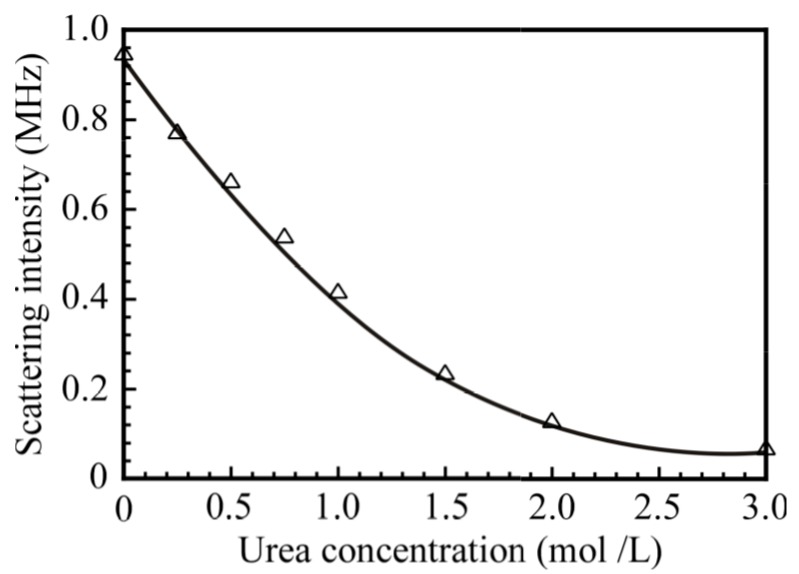
The scattering intensity of the PNaSS_58_–*b*–PAA_125_/PNaSS_58_–*b*–PNIPAM_115_ complex at *C*_p_ = 4.4 g/L in 0.1 M NaCl as a function of the urea concentration.

To confirm the formation of the PNaSS_58_–*b*–PAA_125_/PNaSS_58_–*b*–PNIPAM_115_ complex via hydrogen bonding interactions, urea was added to the mixture of PNaSS_58_–*b*–PAA_125_ and PNaSS_58_–*b*–PNIPAM_115_ in 0.1 M NaCl at pH 3. It is known that urea disturbs hydrogen bonding interactions [25]. Figure 11 shows the light scattering intensity for the PNaSS_58_–*b*–PAA_125_/PNaSS_58_–*b*–PNIPAM_115_ complex as a function of the concentration of urea. The light scattering intensity decreased as the concentration of urea increased from 0 to 3.0 mol/L, and the scattering intensity dropped below 0.1 MHz at 3.0 mol/L urea concentration. These observations are indicative of the complete dissociation of the complex caused by an excess amount of urea. Thus, the complex was confirmed to be formed via hydrogen bonding interactions.

**Figure 12:**
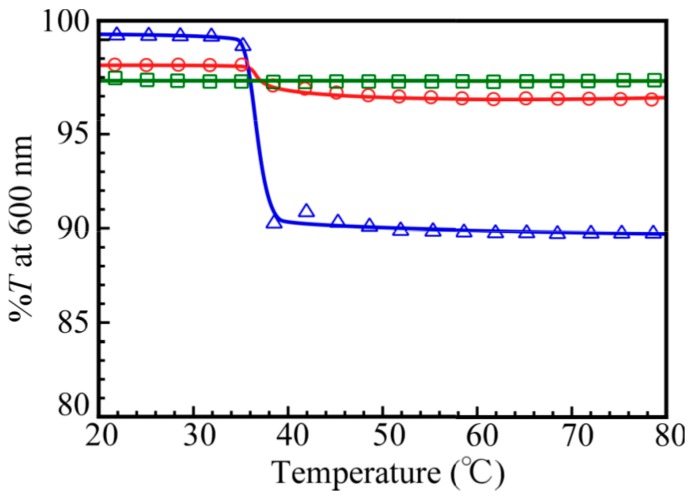
Percent transmittance (%*T*) at 600 nm for 0.1 M NaCl aqueous solutions of PNaSS_58_–*b*–PNIPAM_115_ (△) and a mixture of PNaSS_58_–*b*–PAA_125_ and PNaSS_58_–*b*–PNIPAM_115_ at pH 3 (□) and pH 10 (◯) as a function of solution temperature.

The NIPAM block in the PNaSS_58_–*b*–PNIPAM_115_ diblock copolymer dissolves in water at room temperature, but it separates from aqueous solutions when heated above the lower critical solution temperature (LCST). Figure 12 shows the percent transmittance (*%T*) values monitored at 600 nm for 0.1 M NaCl aqueous solutions of PNaSS_58_–*b*–PNIPAM_115_ and a mixture of PNaSS_58_–*b*–PAA_125_ and PNaSS_58_–*b*–PNIPAM_115_ at pH 3 and 10, as a function of solution temperature. The diblock copolymer, PNaSS_58_–*b*–PNIPAM_115_, exhibited a significant *%T* change at 35–37 °C, which indicates the LCST. The mixture at pH 10 also exhibited a slight *%T* change at 35–37 °C. On the other hand, the mixture at pH 3 did not show any *%T* change in the temperature range from 20 to 80 °C. Ordinarily, the mechanism of LCST for PNIPAM can be explained as follows [26,27]. Below LCST, the PNIPAM chains are hydrated because the pendant amide groups form hydrogen bonding with water molecules, whereas above LCST, molecular motions prevail over the hydrogen bonding interactions resulting in the dehydration of the PNIPAM chains, thus leading to phase separation. Therefore, the PNIPAM chains dehydrated causing phase separation. In the case of the PNaSS_58_–*b*–PAA_125_/PNaSS_58_–*b*–PNIPAM_115_ complex at pH 3, the pendant amide groups in the PNIPAM block interact with the pendant carboxylic acid in the PAA block irrespective of the temperature. Hence, the pendant amide groups in the PNIPAM block are prevented from forming hydrogen bonds with water molecules. Therefore, the LCST of the complex at pH 3 cannot be observed. However, at pH 10, the mixture dissociated and hydrogen bonding interactions between the pendant amide groups in PNaSS_58_–*b*–PNIPAM_115_ and water molecules were formed at low temperature. Hence, the LCST can be observed for the mixture of PNaSS_58_–*b*–PAA_125_ and PNaSS_58_–*b*–PNIPAM_115_ at pH 10, although the decrease in *%T* at the LCST was small comparing to the PNaSS_58_–*b*–PNIPAM_115_ case. This is simply because the concentration of the NIPAM units in the former solution is lower than that in the latter solution.

## 4. Conclusions

The diblock copolymers PNaSS_58_–*b*–PAA_125_ and PNaSS_58_–*b*–PNIPAM_115_ were prepared via RAFT-controlled radical polymerization using a PNaSS_58_ macro–CTA. Both polymerizations proceeded by a controlled mechanism. A mixture of PNaSS_58_–*b*–PAA_125_ and PNaSS_58_–*b*–PNIPAM_115_ formed a water-soluble complex under acidic conditions. The formation of the complex was confirmed by various measurement techniques. The ^1^H NMR data indicated restricted motion of the PNIPAM block at pH 3 in the complex owing to hydrogen bonding interactions between the pendant carboxylic acids in the PAA block and the pendant amide groups in the PNIPAM block. The DLS and SLS data suggested that the PNaSS_58_–*b*–PAA_125_/PNaSS_58_–*b*–PNIPAM_115_ complex was spherical in shape. When urea was added to the complex aqueous solution, the complex dissociated. This observation indicates that the driving force for the formation of the complex is hydrogen bonding interactions. The *%T* data indicated that the LCST of the complex was not observed at pH 3, owing to the complex formation. However, the complex dissociated to a unimer above pH 4.0.

## Figures and Tables

**Table 1:** Degrees of polymerization (DP) of PNaSS, PAA, and PNIPAM blocks, and number-average molecular weights (*M*_n_), and molecular weight distributions (*M*_w_/*M*_n_) of the diblock copolymers.

Samples	DP of PNaSS ^a^	DP of PAA ^b^	DP of PNIPAM ^b^	*M*_n_(theo) ^c^ × 10^−4^	*M*_n_(NMR) ^b^ × 10^−4^	*M*_n_(GPC) ^a^ × 10^−4^	*M*_w_/*M*_n_^a^
PNaSS_58_–*b*–PAA_125_	58	125		2.07	2.12	3.51	1.18
PNaSS_58_–*b*–PNIPAM_115_	58		115	2.27	2.52	1.21	1.39

^a^ Estimated from gel permeation chromatography (GPC) eluted with a phosphate buffer solution at pH 9 containing 10 vol % acetonitrile; ^b^ Estimated from ^1^H NMR; ^c^ Calculated from Equation (4).

**Table 2:** Light scattering data for the PNaSS_58_–*b*–PAA_125_/PNaSS_58_–*b*–PNIPAM_115_ complex.

pH	*R*_h_^a^ (nm)	*M*_w_(SLS) ^b^ × 10^−5^	*R*_g_^b^ (nm)	*R*_g_/*R*_h_	*N_agg_* ^c^
3	15.0	9.23	12.9	0.86	46
10	3.7	0.20	13.6	3.7	1

^a^ Estimated from dynamic light scattering (DLS); ^b^ Estimated from static light scattering (SLS); ^c^ Aggregation number calculated from *M*_w_ of the aggregate determined at pH 3 and *M*_w_ of the corresponding unimer at pH 10 determined from SLS.
